# Sunitinib-Induced Congestive Heart Failure in a Patient with Gastrointestinal Stromal Tumor

**DOI:** 10.34172/aim.2022.64

**Published:** 2022-06-01

**Authors:** Wala Ben Kridis, Sonda Masmoudi, Salma Ben Charfeddine, Afef Khanfir

**Affiliations:** ^1^Department of Medical Oncology, Habib Bourguiba Hospital, University of Sfax, Tunisia; ^2^Department of Cardiology, Hedi Chaker Hospital, University of Sfax, Tunisia

**Keywords:** Cardiotoxicity, Follow up, Heart failure, Rechallenge sunitinib, Treatment

## Abstract

Common cardiovascular toxicities of sunitinib mainly include hypertension, QT prolongation, left ventricular dysfunction (LVD) and less frequently, congestive heart failure (CHF). Here, we report the case of a 67-year-old woman who developed heart failure after 24 months of sunitinib. Our case highlights the importance of strict and regular cardiovascular monitoring during sunitinib. It also shows that the reintroduction of sunitinib with maintaining heart failure treatment can be safe. The exact mechanisms of this cardiotoxicity have not been understood. There is no protective therapy available. Therefore, further investigations are needed in these areas. Medical specialists who prescribe and treat patients with sunitinib should be aware of the possible occurrence of these conditions and perform regular checkup of sunitinib-treated patients.

## Introduction

 Tyrosine-kinase inhibitors (TKIs) are targeted therapies. They inhibit kinases that are mutated or over-expressed in tumor cells.^1^ However, these drugs can also inhibit some kinases that are expressed normally by non-cancer cells, which can lead to toxicities.^2^ Sunitinib is a multitargeted TKI. It exerts its effects majorly by blocking the vascular endothelial growth factor (VEGF).^2^ It additionally inhibits a wide range of molecules such as c-kit (CD117), platelet-derived growth factor receptors, colony-stimulating factor 1 receptor, RET and FLT-3 kinases.^3^ Common cardiovascular toxicities of sunitinib include hypertension, QT prolongation, left ventricular dysfunction (LVD) and less frequently, congestive heart failure (CHF).^4^ CHF is defined by decline of left ventricular ejection fraction (LVEF) below normal values, with symptoms of heart failure (i.e. dyspnea, orthopnea, fatigue jugular venous distention and/or pedal edema) and documented pulmonary edema on chest X-ray or symptoms responding to classic treatments of CHF.^4^ Approximately, 4.3% of patients treated with sunitinib may develop a decline in LVEF or CHF.^4^ Here, we report the case of a 67-year-old woman who developed CHF after 24 months of sunitinib.

## Case Report

**Figure 1:**
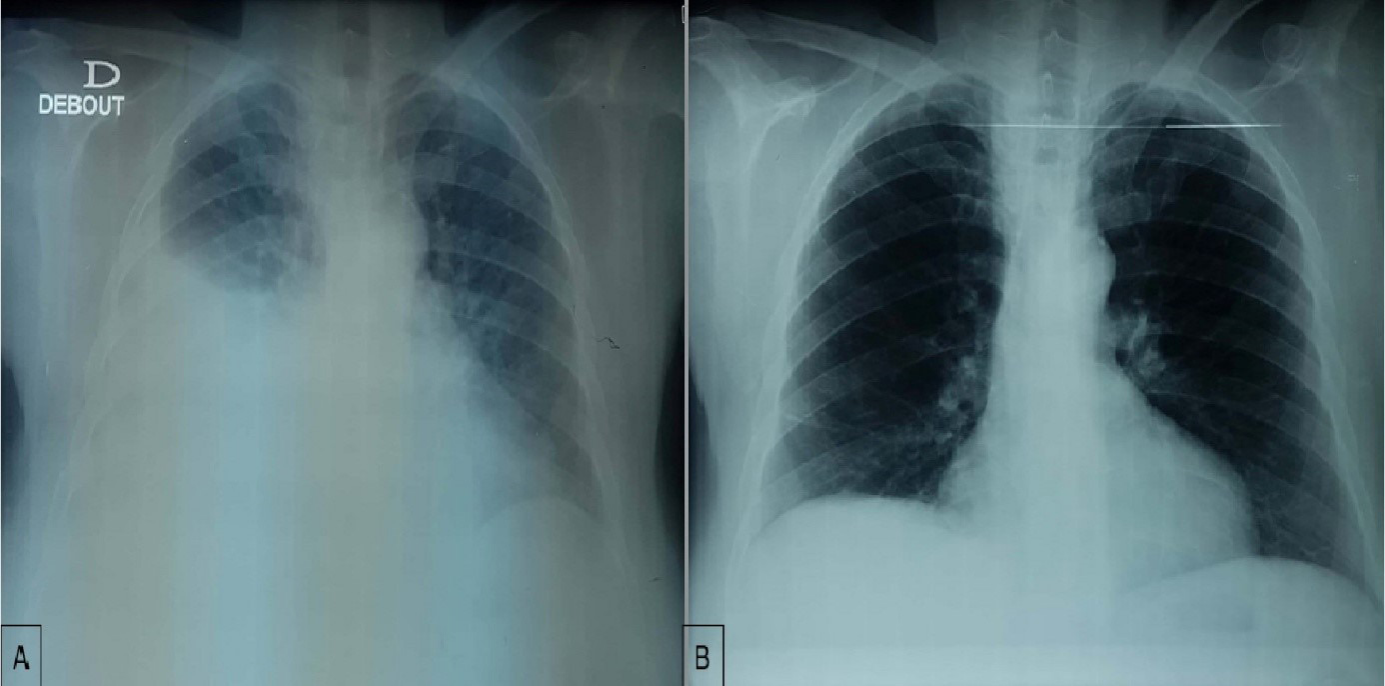
**(A)** Radiography on admission to our department showing right pleural effusion. **(B)** Chest x-ray after one week of heart failure treatment and stopping sunitinib.

**Figure 2:**
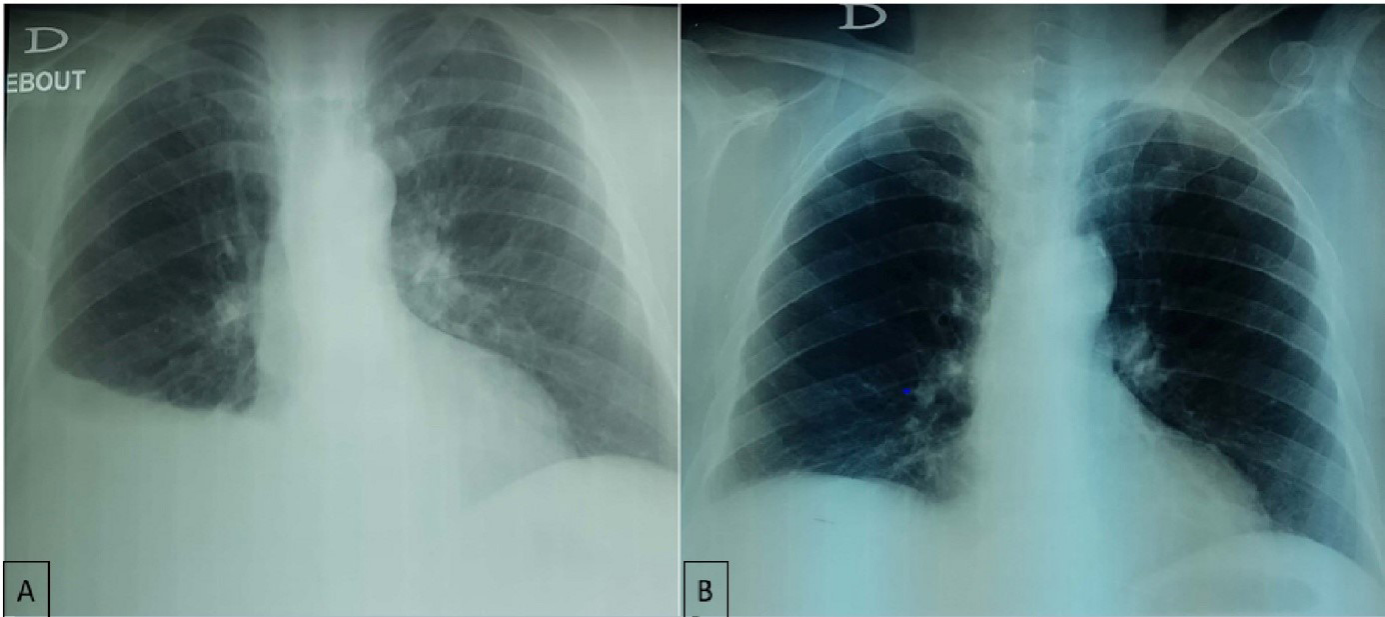
**(A)** Chest x-ray after sunitinib reintroduction without heart failure treatment. **(B)** Chest x-ray after sunitinib with maintaining heat failure treatment.

 A 67-year-old woman with no medical history was diagnosed 4 years earlier with an unresectable mesenteric gastrointestinal stromal tumor (GIST). She initially underwent systemic treatment with Imatinib 400 mg/d for 12 months and then 800mg/day for 6 months. Because of disease progression, she received sunitinib at a dose of 50mg/day for 4 weeks followed by 2 weeks of rest. The LVEF before initiation of sunitinib was normal. Initially, the treatment was well tolerated beyond a well-balanced hypothyroidism under levothyroxine. Then, she presented a grade 2 hand-foot syndrome with digestive intolerance. So, we administered sunitinib as 2 weeks on followed by 1 week off. The patient was monitored for her disease with CT every 3 months. The disease was stable with good tolerance. After 24 months of treatment, the patient presented, in emergency, for dyspnea. The COVID-19 PCR test was negative. The blood pressure was normal. Lower limbs edema was noted. Electrocardiogram found a right bundle branch block. Chest X-ray showed right pleural effusion (Figure 1A). There was no evidence of disease progression on CT. The thyroid test was normal. Analysis of the pleural puncture liquid revealed a transudate liquid free of malignant cells on cytological examination. This dyspnea was responsive to diuretics. Echocardiogram showed a reduction of LVEF at 43%. CHF was then diagnosed. Sunitinib was discontinued. Diuretics, beta-blocker and an angiotensin-converting enzyme inhibitor were administrated with the disappearance of dyspnea, regression of pleural effusion on chest X-ray (Figure 1B) and normal LVEF. Sunitinib was reintroduced while maintaining the treatment for heart failure. However, the patient forgot to take heart failure treatment. Chest X-ray showed the recurrence of pleural effusion (Figure 2A) with a decrease in LVEF. After regular observation of heart failure treatment during sunitinib, chest X-ray (Figure 2B) and echocardiogram became normal. Therefore, the diagnosis of grade 3 cardiotoxicity secondary to sunitinib was retained. Weekly cardiac monitoring with echocardiogram was performed. LVEF is still stable one month after reintroduction of sunitinib with no evidence of pleural effusion.

## Discussion

 CHF is an impairment of the cardiac pump that may be caused by structural or functional heart anomalies.^4^ The most common causes of this disorder are ischemic disease, hypertension, valvular disease and idiopathic dilated cardiomyopathy. Several other causes of CHF have been identified such as obesity, diabetes mellitus, atrial fibrillation, hyperlipidemia, and hypothyroidism and hyperthyroidism.

 In our department, among 16 patients treated with sunitinib, only one patient presented the symptoms of CHF (6.25%). This was the first case in our institution. In a retrospective study by Chu et al, 8% presented with CHF.^5^ Totally, 28% of patients treated with sunitinib had absolute reductions in LVEF. Diverging findings were reported by Motzer et al who reported a 10% rate of reduction in LVEF in renal-cell carcinoma patients treated with sunitinib for a median of 6 months.^6^ However, no patient developed heart failure. Another retrospective study by Khakoo et al on 224 patients treated with sunitinib^7^ showed that six patients (2.7%) developed CHF. An overview of cardiovascular toxicity of TKI from 113 systematic reviews published by Van Leeuwen et al in 2020 found that 4.3% LVEF decline or CHF during treatment with sunitinib.^4^

 The exact mechanism of CHF associated with sunitinib remains unclear. Proposed mechanisms include inhibition of platelet-derived growth factor receptor, VEGF, or KIT receptors, as well as caspase-activated mitochondrial apoptosis that leads to direct injury of myocytes.^8^ In addition, this cardiotoxicity may be exacerbated by hypertension and hypothyroidism.^8^ In our case, there was no evidence of hypertension throughout the treatment period. However, the patient was treated for hypothyroidism secondary to sunitinib. CHF and LVEF induced by sunitinib are generally reversible when sunitinib is stopped and the classic treatment of CHF is introduced (grade 3 toxicity),^5^ as in our case. In the retrospective review by Chu et al, after interruption or reduction of sunitinib doses, the symptoms improved in five of the six patients with CHF, and they resumed sunitinib therapy without incident.^5^ This review provided evidence that TKIs can be tried again after heart function returns to normal, especially if the patient had a previous response to therapy. In our case, the reintroduction of sunitinib with maintaining heart failure treatment was safe.

 In conclusion,sunitinib has been widely applied in several cancers with encouraging results. Data is emerging about its cardiotoxicity. The decrease in the LVEF is an infrequent adverse effect of sunitinib. It is generally reversible when appropriate treatments are delivered. However, the fear is that symptomatic heart failure may cause significant morbidity. Hence, strict and regular cardiovascular monitoring is mandatory during treatment with sunitinib. The exact mechanisms of this cardiotoxicity have not been understood. There is no protective therapy available. Therefore, further investigations are needed in these areas.
